# Tuning the Electronic Properties of Cu_m_Ag_n_ Bimetallic Clusters for Enhanced CO_2_ Activation

**DOI:** 10.3390/ijms252212053

**Published:** 2024-11-09

**Authors:** Turki Alotaibi, Moteb Alotaibi, Fatimah Alhawiti, Nawir Aldosari, Majd Alsunaid, Lama Aldawas, Talal F. Qahtan, Ali K. Ismael

**Affiliations:** 1 Physics Department, College of Science, Jouf University, Sakakah 11942, Saudi Arabia; tbotaibi@ju.edu.sa; 2 Department of Physics, College of Science and Humanities, Prince Sattam Bin Abdulaziz University, Al-Kharj 11942, Saudi Arabia; 442050824@std.psau.edu.sa (N.A.); 442051726@std.psau.edu.sa (M.A.); 442051238@std.psau.edu.sa (L.A.); t.qahtan@psau.edu.sa (T.F.Q.); 3 Department of Physics, University College of Taraba, Taif University, Taraba 21944, Saudi Arabia; frhawiti@tu.edu.sa; 4 Department of Physics, Lancaster University, Lancaster LA1 4YB, UK

**Keywords:** bimetallic clusters, CO_2_ activation, DFT, sustainability, climate change

## Abstract

The urgent demand for efficient CO_2_ reduction technologies has driven enormous studies into the enhancement of advanced catalysts. Here, we investigate the electronic properties and CO_2_ adsorption properties of Cu_m_Ag_n_ bimetallic clusters, particularly Cu_4_Ag_1_, Cu_1_Ag_4_, Cu_3_Ag_2_, and Cu_2_Ag_3_, using generalized gradient approximation (GGA)/density functional theory (DFT). Our results show that the atomic arrangement within these clusters drastically affects their stability, charge transfer, and catalytic performance. The Cu_4_Ag_1_ bimetallic cluster emerges as the most stable structure, revealing superior charge transfer and effective chemisorption of CO_2_, which promotes effective activation of the CO_2_ molecule. In contrast, the Cu_1_Ag_4_ bimetallic cluster, in spite of comparable adsorption energy, indicates insignificant charge transfer, resulting in less pronounced CO_2_ activation. The Cu_3_Ag_2_ and Cu_2_Ag_3_ bimetallic clusters also display high adsorption energies with remarkable charge transfer mechanisms, emphasizing the crucial role of metal composition in tuning catalytic characteristics. This thorough examination provides constructive insights into the design of bimetallic clusters for boosted CO_2_ reduction. These findings could pave the way for the development of cost-effective and efficient catalysts for industrial CO_2_ reduction, contributing to global efforts in carbon management and climate change mitigation.

## 1. Introduction

Carbon dioxide (CO_2_) is a primary greenhouse gas responsible for global warming and climate change [1,2,3,4]. Its increasing concentration in the atmosphere, predominantly due to anthropogenic activities such as fossil fuel combustion and deforestation, has created an urgent demand for effective mitigation strategies. One promising strategy to address this issue is the catalytic conversion of CO_2_ into valuable chemicals, such as ethylene (C_2_H_4_), formic acid (CH_2_O_2_), and ethanol (C_2_H_6_O), among other hydrocarbons [5,6]. This method not only helps decrease CO_2_ atmospheric levels but also yields products with broad industrial applications [7,8]. Moreover, CO_2_ conversion can be conducted under ambient conditions with electricity generated from renewable sources such as wind, solar, or hydropower, making it an environmentally friendly solution [7,8].

Over the years, significant research efforts have been dedicated to developing effective catalysts for the electrochemical CO_2_ reduction reaction (CO_2_RR) [9,10]. Early studies on CO_2_RR catalysts mainly focused on noble metal-based catalysts, including platinum (Pt) [11], rhodium (Rh) [12], iridium (Ir) [13], palladium (Pd) [14], and gold (Au) [15]. Although these catalysts demonstrate high efficiency, their limited availability and high cost hinder scalability [11,12,13,14,15]. Consequently, there is an increasing need for earth-abundant metal-based catalysts that can sustainably and effectively transform CO_2_ [9,10,16].

Among these alternatives, copper (Cu) has gained attention as it facilitates the conversion of CO_2_ into a wide range of products, including hydrocarbons and alcohols [16,17,18,19]. Research by Dong et al. revealed that Cu_79_ clusters demonstrate a reduced energy barrier for CO_2_ reduction to CO, attributable to their high surface-to-volume ratio and coordinatively unsaturated atoms [20]. Similarly, smaller clusters like Cu_4_ and Cu_29_ have shown potential for CO_2_ conversion to methanol (CH_3_OH) at low activation energies [21,22,23,24]. Despite these advancements, current Cu-based catalysts often require high overpotentials for practical applications, resulting in low selectivity for desired products [25].

To address these challenges, recent studies have explored combining Cu with other metals to enhance CO_2_RR performance. Bimetallic systems, particularly Cu-Ag combinations, are known for their synergistic interactions, creating new active sites that improve catalytic efficiency [26]. Ag has shown particular promise due to its selectivity in CO_2_ reduction at moderate overpotentials, effectively suppressing competing hydrogen evolution reactions (HER) [27,28]. Studies have demonstrated that incorporating Ag with Cu not only enhances catalytic efficiency but also provides an economically feasible alternative to noble metals like Au and Pt [29,30]. The significance of Cu-Ag bimetallic clusters is further supported by recent experimental findings. For instance, Huang et al. observed that Cu-Ag nanowire interfaces increase methane production during CO_2_ reduction, achieving a Faraday efficiency of 72% [31]. This highlights the importance of understanding how varying Cu-Ag ratios affect CO_2_ adsorption and activation at the atomic level, as it can guide the design of optimized bimetallic catalysts [32,33,34].

Although research on CO_2_/Cu-Ag bimetallic clusters is inadequate [35,36], there is also a lack of detailed understanding of the atomic-level morphology of Cu-Ag nanoalloys and their interactions with CO_2_. To the best of our knowledge, the trapezoidal configuration of Cu_m_Ag_n_ clusters (where m + n = 5) with varying compositions of Ag atoms within the same system and their susceptibility to CO_2_ adsorption have not been tackled yet. Hence, it is compelling to examine the influence of Ag mixing on the stability and electronic properties of Cu clusters and their interactions with CO_2_ molecules. Identifying the most stable configuration for bimetallic clusters is a challenging task caused by the intricate nature of their structural complexity. Thus, it is necessary to implement the efficient density functional theory (DFT) method to investigate the potential configurations of clusters/alloys that are close to the ground state.

This study aims to address these gaps by employing DFT calculations to systematically investigate the stability, electronic properties, and CO_2_ adsorption behaviors of the Cu_m_Ag_n_ bimetallic cluster. By examining different compositions and configurations, we seek to identify the most stable structures and understand the mechanisms behind their interaction with CO_2_. The insights gained from this study could guide the design of more efficient and selective bimetallic catalysts, contributing to the development of sustainable CO_2_ reduction technologies and offering potential pathways for the development of scalable catalytic solutions.

**Figure 1:**
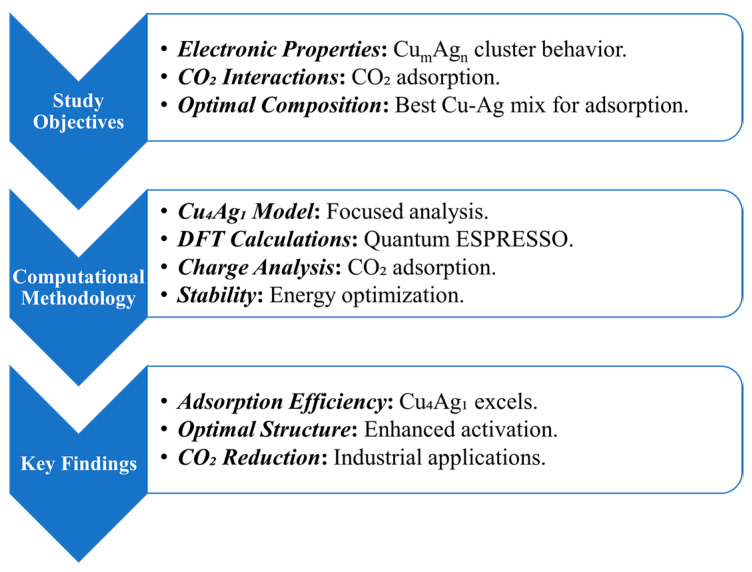
Flowchart of study objectives, DFT-based methodology, and key findings, emphasizing the efficiency of the Cu_4_Ag_1_ cluster in CO_2_ reduction.

To provide a comprehensive overview of the objectives of the study and methodological approach, Figure 1 presents a visual summary that clarifies the key stages of our research. This study begins with the identification of the optimal compositions of Cu_m_Ag_n_ bimetallic clusters, specifically targeting electronic properties favorable for CO_2_ activation. The flowchart outlines the step-by-step computational methodology employed, including the use of density functional theory (DFT) for structural and electronic analysis. By focusing on specific clusters, such as Cu_4_Ag_1_, the study aims to quantify charge transfer and adsorption energies, ultimately pinpointing the cluster composition that maximizes CO_2_ adsorption efficiency. These findings are particularly relevant for advancing catalytic applications in industrial CO_2_ reduction, providing a basis for future experimental studies.

## 2. Results and Discussion

### 2.1. Cu_4_Ag_1_ and Cu_1_Ag_4_ Bimetallic Clusters

**Figure 2:**
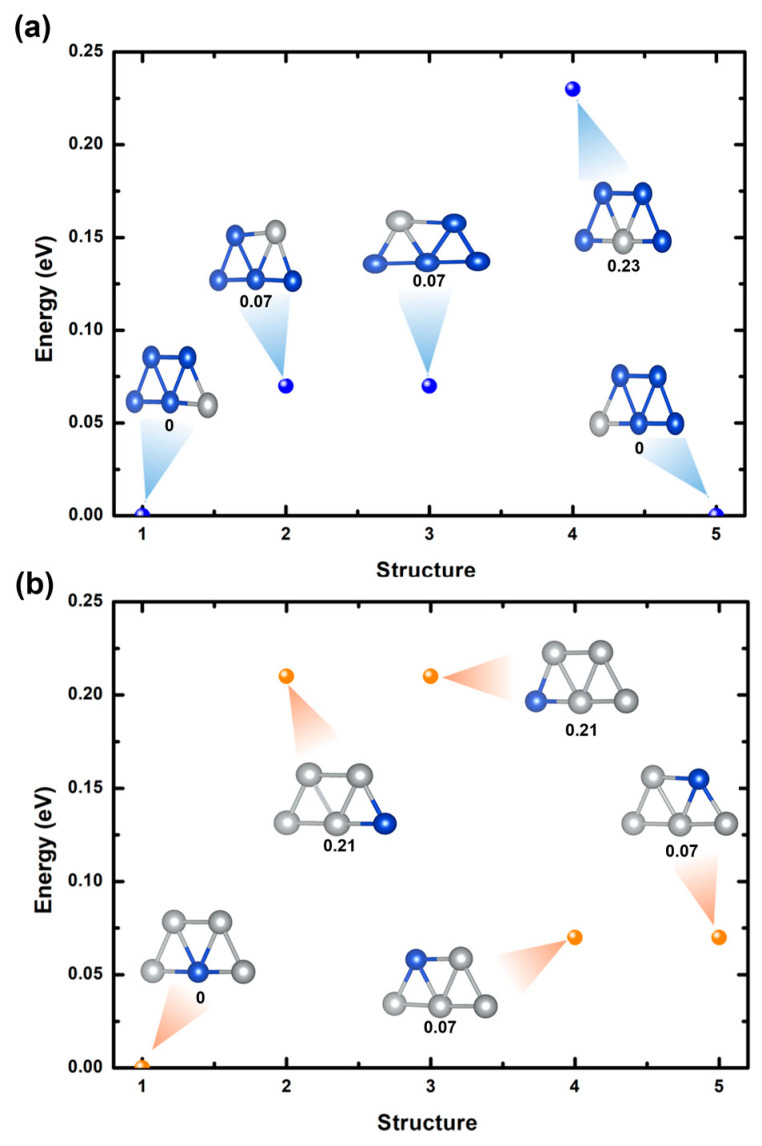
(**a**) Relative energies of optimized Cu_4_Ag_1_ bimetallic clusters by varying sites of Ag atom. (**b**) Relative energies of optimized Cu_1_Ag_4_ bimetallic clusters by varying sites of Cu atom. Blue and gray balls denote copper and silver atoms, respectively.

Figure 2a,b depict five optimized Cu_4_Ag_1_ trapezoidal bimetallic clusters and five optimized Cu_1_Ag_4_ trapezoidal bimetallic clusters using GGA/PBE. For both cases, the ground states of these clusters are two-dimensional trapezoidal configurations, consistent with previous studies [37]. For Cu_4_Ag_1_ clusters, the most stable bimetallic cluster is observed when the Ag atom is positioned at the bottom corners of the cluster (structures 1 and 5 in Figure 2a). This result implies that the bottom corner sites promote a more favorable electronic environment for the Ag atom, possibly owing to enhanced bonding interactions with the neighboring Cu atoms. The metastable state takes place when the Ag atom is located at the top corners of the cluster (structures 2 and 3 in Figure 2a), with an energy difference of approximately 0.07 eV compared to the most stable cluster. This metastable state indicates that the top corner positions are less favorable but still within a relatively low energy range, signifying potential flexibility in the structural variation of the clusters. The least stable structure is recognized when the Ag atom occupies the center of the cluster (structures 4 in Figure 2a), revealing an energy difference of 0.23 eV. Figure 2a also confirms that changing a Cu atom with an Ag atom at various sites within the cluster induces different degrees of deformation. This deformation is ascribed to changes in bond length, aligning with prior research [38]. Moreover, the calculated average bond length of Cu-Cu is 2.38 Å and of Cu-Ag is 2.51 Å, which is close to the bulk value of 2.53 Å [39].

For Cu_1_Ag_4_ clusters, the most favorable configuration for the bimetallic cluster is achieved when the Cu atom is situated at the center of the cluster, as observed in structure 1 of Figure 2b. This stability is consistent with previous studies [37,40,41]. The observed stability implies that the central sites provide a more favorable electrical environment for the Cu atom, possibly due to increased bonding interactions with the surrounding Ag atoms. A metastable condition occurs when the Cu atom is positioned at the upper corners of the cluster (structures 4 and 5 in Figure 2b), with an energy difference of about 0.07 eV compared to the most stable cluster. This indicates that although the top corner positions are not immensely favorable, they nevertheless fall within a low energy range, demonstrating a certain level of structural adaptability. The most unstable cluster is noted when the Cu atom is positioned in the cluster’s bottom corners (structures 2 and 3 in Figure 2b), inducing an energy difference of 0.21 eV. Moreover, Figure 2b reveals that replacing an Ag atom with a Cu atom at different sites of the cluster leads to different levels of distortion. The distortions observed are attributed to variations in the length of chemical bonds. In addition, the average bond lengths computed for Ag-Ag and Cu-Ag are 2.66 Å and 2.55 Å, respectively.

**Figure 3:**
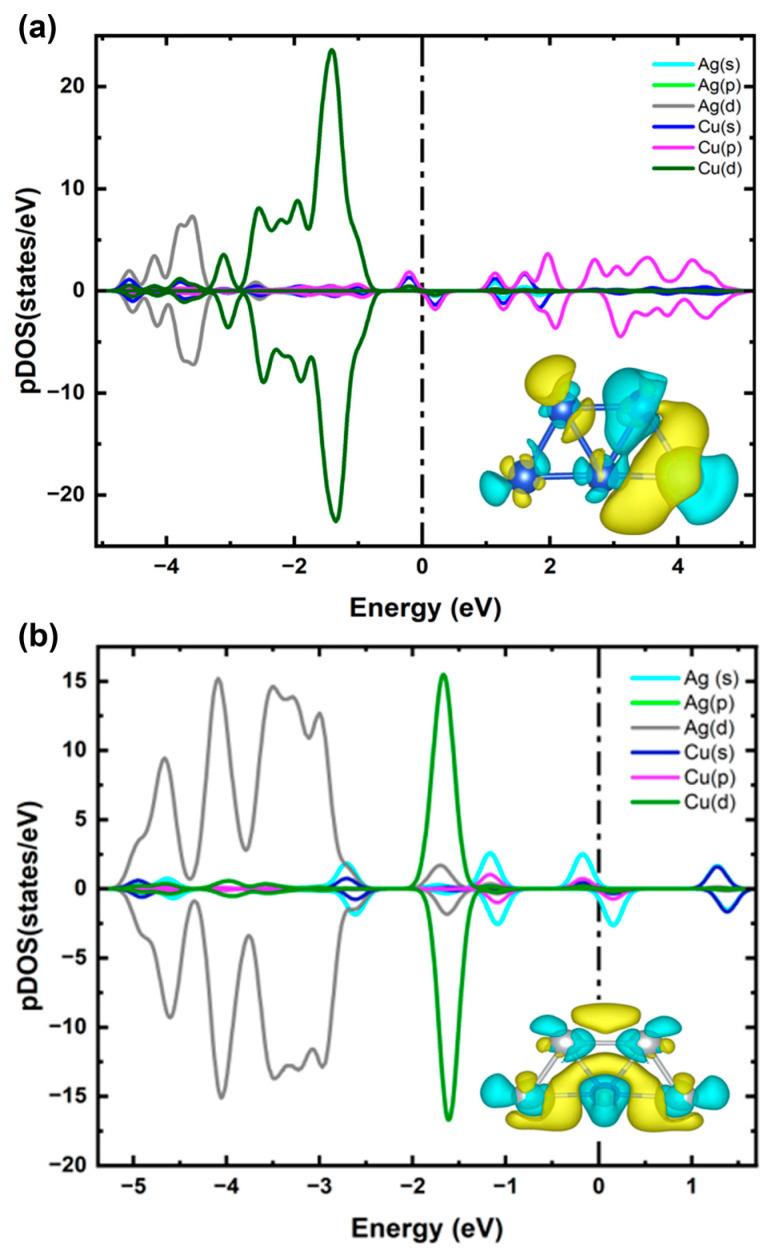
(**a**) pDOS and CDD of Cu_4_Ag_1_ bimetallic cluster. (**b**) pDOS and CDD of Cu_1_Ag_4_ bimetallic cluster. The vertical line shows the Fermi level, and blue and yellow clouds indicate the negative and positive potentials.

To clarify the charge transfer mechanism and electronic properties of the lowest-energy configuration of Cu_4_Ag_1_ and Cu_1_Ag_4_ clusters (see structures 1 in Figure 2a,b), we applied the Bader charge analysis technique, calculated the pDOS, and presented the CDD, as illustrated in Figure 3. Bader charge calculations demonstrate that for Cu_4_Ag_1_ cluster, the Ag atom gains ~0.18 e^−^ from the Cu atoms, while for Cu_1_Ag_4_ cluster, the Ag atoms gains ~0.22 e^−^ from the Cu atom, a phenomenon clearly proven in the CDD plot. The driving force behind this charge transfer is the difference in Pauling electronegativity between Ag (1.93) and Cu (1.90) [39]. Though the variation in electronegativity is relatively small, it is sufficient to induce an obvious electron transfer, revealing the sensitivity of these clusters to subtle electronic changes. The pDOS calculations further complement these analyses by illustrating the electronic states contributing to the bonding and stability of the cluster. For the Cu_4_Ag_1_ cluster, it is evidently observed that the states range between −3.3 eV and −0.8 eV and are dominated mainly by the d orbitals of Cu atoms, with the insignificant contribution of the d orbitals of the Ag atom at around −2.7 eV. The contribution of d orbitals of the Ag atom is more pronounced in the energy range between −4.8 eV and −3.35 eV, with a minor hybridization of the d orbitals of Cu atoms at nearly −3.9 eV.

For the Cu_1_Ag_4_ cluster, it is evident that the states within the energy range of −2 eV to −1.3 eV are mainly dominated by the d orbitals of Cu atoms, with a slight contribution from the d orbitals of the Ag atom at around −1.6 eV. In the energy range of −5.2 eV to −2.5 eV, the d orbitals of the Ag atom are more explicit, displaying minor hybridization with the d and s orbitals of the Cu atom near −3.8 eV and −2.7 eV, respectively. The incorporation of Cu into the Ag cluster leads to an improved occupation of the s orbitals of the Ag atoms, as clearly apparent by the overlapped state at −1 eV in Figure 2b. This occurrence likely implies that Cu doping facilitates the d-electrons transfer to the Ag atoms within the Cu_1_Ag_4_ cluster. This electron transfer was also found in the Ag_12_Cu cluster [42].

For both clusters, doping an Ag atom in the Cu cluster and vice versa modifies the electronic structure, as evidenced by the changes in the DOS near the Fermi level (see Figure 3b). In particular, there is obvious hybridization between the d orbitals and sp orbitals near the Fermi level in the Cu atom and the p orbitals in the Ag atoms (see Figure 3a). Similarly, the Ag atoms display boosted overlap of their s orbitals with the sp hybridized orbitals of the Cu atom near the Fermi level (see Figure 3b). This leads to electron transfer from the Cu atom to the Ag atoms in both Cu_4_Ag_1_ and Cu_1_Ag_4_ clusters, resulting in a substantial splitting between the spin-up and spin-down DOS, which is consistent with previous reports [43]. This hybridization can improve the catalytic properties of the clusters by creating active sites with altered electronic environments, which are necessary for facilitating various chemical reactions.

### 2.2. Cu_3_Ag_2_ and Cu_2_Ag_3_ Bimetallic Clusters

**Figure 4:**
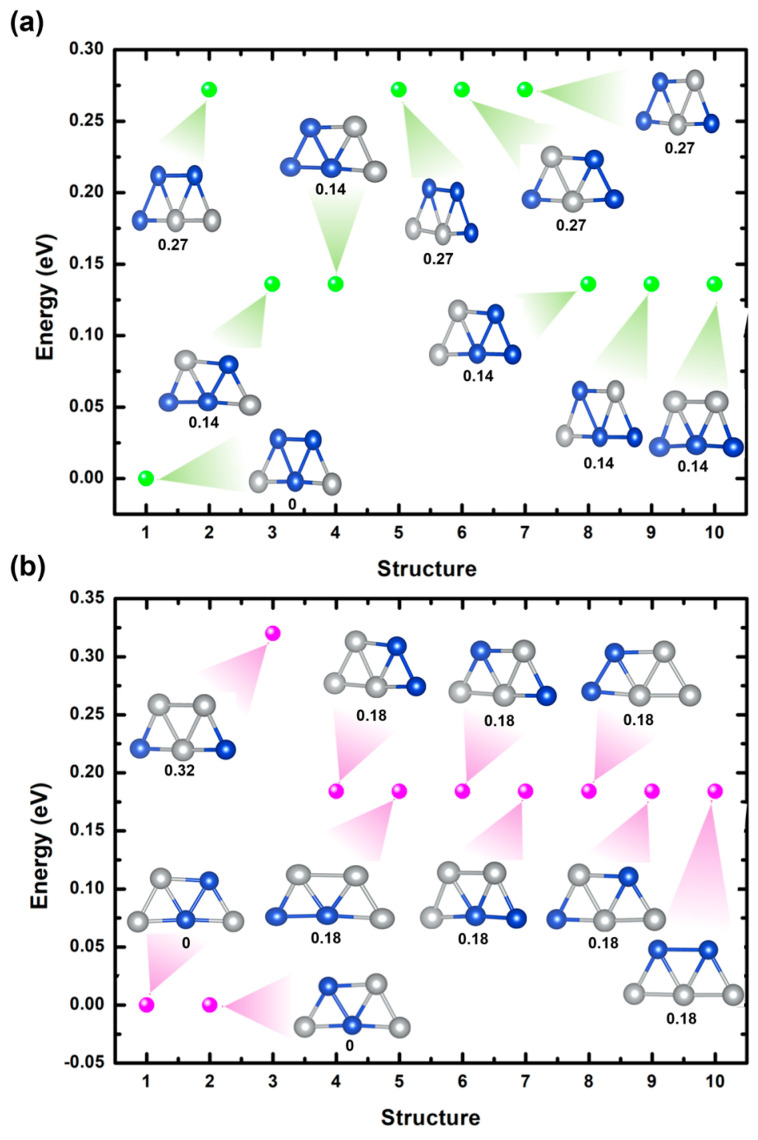
(**a**) Relative energies of optimized Cu_3_Ag_2_ bimetallic clusters by varying sites of the Ag atom. (**b**) Relative energies of the optimized Cu_2_Ag_3_ bimetallic clusters by varying sites of the Cu atom.

Figure 4a,b illustrate ten optimized trapezoidal bimetallic clusters of Cu_3_Ag_2_ and Cu_2_Ag_3_, respectively, utilizing GGA/PBE methodology. In both systems, the clusters’ ground states reveal two-dimensional trapezoidal structures, corroborating the results of earlier research [37,40]. For Cu_3_Ag_2_ clusters, the most stable cluster is achieved when two Ag atoms are located at the bottom corners of the cluster (structures 1 in Figure 4a). This stability possibly arises from the bottom corner sites providing a more favorable electronic environment for the Ag atoms, potentially due to improved bonding interactions with the neighboring Cu atoms. A metastable state is noted for structures 3, 4, 5, 9, and 10 (see Figure 4a), with an energy difference of around 0.14 eV compared to the most stable cluster. The least stable structure arises for structures 2, 6, 7, and 8 (see Figure 4a), with an energy difference of 0.27 eV. Figure 4a further demonstrates that substituting of tow Cu atoms with Ag atoms at different sites within the cluster results in varying degrees of deformation, ascribed to alterations in bond lengths. Moreover, the calculated average bond lengths of the most stable cluster are 2.40 Å for Cu-Cu and 2.52 Å for Cu-Ag.

For Cu_2_Ag_3_ clusters, the most favorable arrangement is obtained when two Cu atoms are positioned within the center and top corners of the cluster, as depicted in structures 1 and 2 of Figure 4b. The observed stability implies that these sites offer a more favorable electronic environment for the Cu atom, likely due to enhanced bonding interactions with the surrounding Ag atoms. A metastable state is found for structures 4, 5, 6, 7, 8, 9, and 10 (see Figure 4b), with an energy difference of nearly 0.18 eV compared to the most stable cluster. This signifies that, while the bottom corner positions are less favorable, they remain within a low energy range, showing some degree of structural adaptability. The least stable cluster is observed when the Cu atoms are located at the bottom corners of the cluster (structure 3 in Figure 4b), resulting in an energy difference of about 0.32 eV. Moreover, Figure 4b shows that replacing Ag atoms with Cu atoms at various sites of the cluster induces different levels of distortion. These distortions are caused by changes in bond lengths. Furthermore, the computed average bond lengths for the lowest energy state are 2.62 Å, 2.42 Å, and 2.53 Å for Ag-Ag, Cu-Cu, and Cu-Ag, respectively.

**Figure 5:**
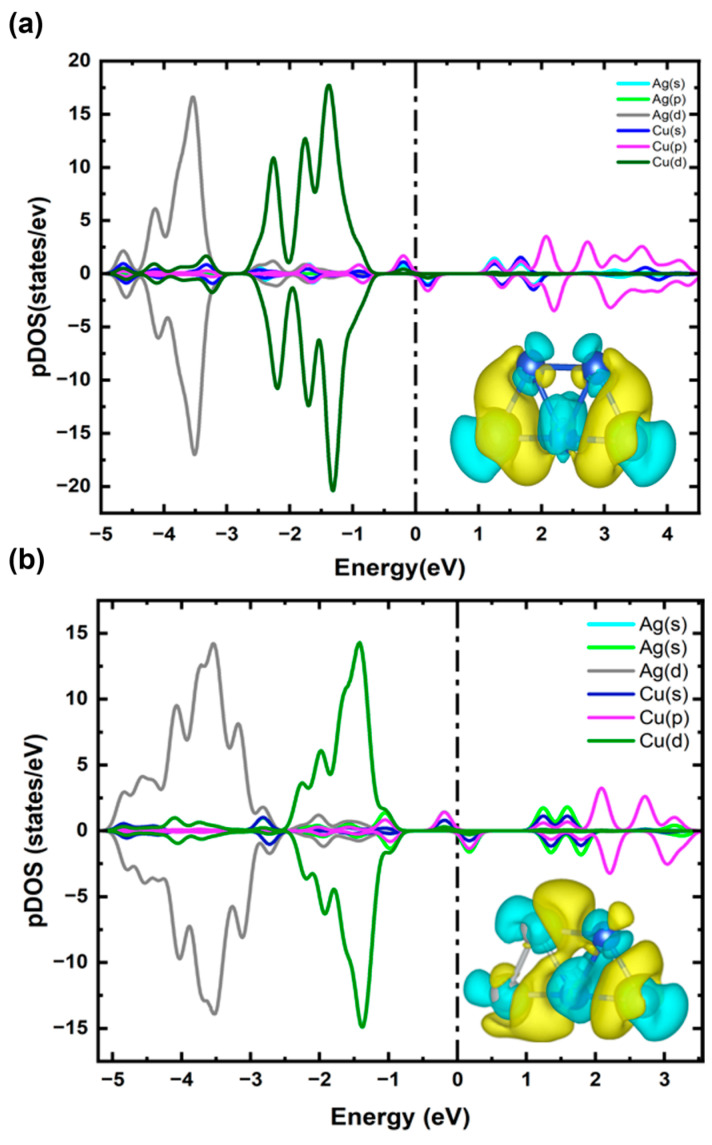
(**a**) pDOS and CDD of the Cu_3_Ag_2_ bimetallic cluster. (**b**) pDOS and CDD of the Cu_2_Ag_3_ bimetallic cluster. The vertical line shows the Fermi level, and blue and yellow clouds indicate the negative and positive potentials.

To describe the charge transfer mechanism and the electronic properties of the lowest-energy structures of Cu_3_Ag_2_ and Cu_2_Ag_3_ clusters (see structures 1 in Figure 4a,b) we employed Bader charge analysis, calculated the pDOS, and illustrated the CDD, as presented in Figure 5. The Bader charge calculations indicate that in the Cu_3_Ag_2_ cluster, the Ag atoms gain approximately 0.30 e^−^ from the Cu atoms, whereas in the Cu_2_Ag_3_ cluster, the Ag atoms gain about 0.41 e^−^ from the Cu atoms. This phenomenon is clearly observed in the charge density difference plots seen in Figure 5a,b. The pDOS calculations offer further insights by identifying the electronic states that contribute to the bonding and stability of the clusters. In the Cu_3_Ag_2_ cluster, the energy range from −2.7 eV to −0.7 eV is predominantly influenced by the d orbitals of Cu atoms, with a modest contribution from the d states of the Ag atom around −2.1 eV. The Ag atom’s d orbitals are particularly prominent in the energy range from −4.8 eV to −3.1 eV, with a slight hybridization of the Cu atoms’ d orbitals.

In terms of the Cu_3_Ag_2_ cluster, the energy range from −2.5 eV to −0.8 eV is mainly dominated by the d orbitals of Cu atoms, with a small contribution from the d states of the Ag atom around −2.0 eV. The d states of the Ag atom become more prominent in the energy range from −5.0 eV to −2.5 eV, indicating modest hybridization with the d and s orbitals of the Cu atom around −4.0 eV and −2.7 eV, respectively. The incorporation of Cu into the Ag cluster results in a greater filling of the s orbitals of Ag atoms, as evidenced by the overlapping state at −1.1 eV in Figure 5b. This suggests that the addition of Cu facilitates the transfer of d-electrons to the Ag atoms in the Cu_2_Ag_3_ cluster. The electronic structure of both clusters is influenced by the doping of Ag and Cu atoms in the Cu cluster and vice versa. This effect is evident from the variations in the DOS near the Fermi level. Specifically, there is clear hybridization between the d states and sp levels near the Fermi level within the Cu atom, as well as the p orbitals within the Ag atoms (see Figure 5a). Similarly, the Ag atoms, which have neighboring atoms, demonstrate pronounced overlap of their s orbitals with the sp hybridized orbitals of the Cu atom around the Fermi level (see Figure 5b). This interaction causes the transfer of charge from the Cu atom to the Ag atoms in both Cu_4_Ag_1_ and Cu_1_Ag_4_ clusters, resulting in a significant separation between the spin-up and spin-down DOS. This observation is consistent with previous studies [43]. The hybridization can enhance the catalytic properties of the clusters by producing active sites with modified electronic environments, which are essential for facilitating several chemical reactions.

### 2.3. Adsorption of CO_2_ on Cu_4_Ag_1_ and Cu_1_Ag_4_ Clusters

**Figure A1:**
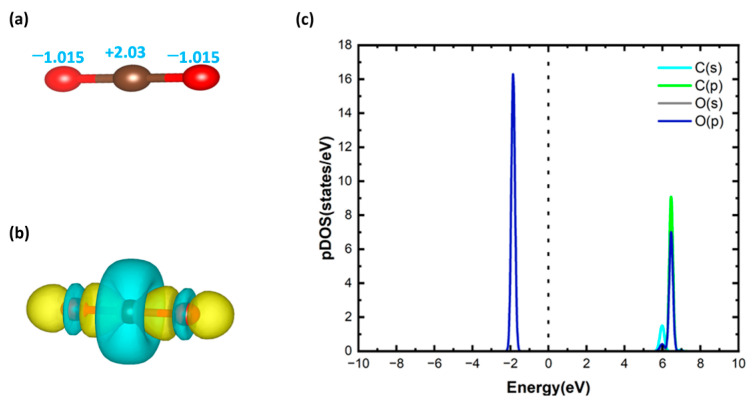
(**a**) Optimized structure of the isolated CO_2_ molecule. (**b**) Charge density difference of CO_2_. (**c**) Partial density of states of CO_2_. Note: blue numbers represent the amount of electrons on each atom.

**Figure A2:**
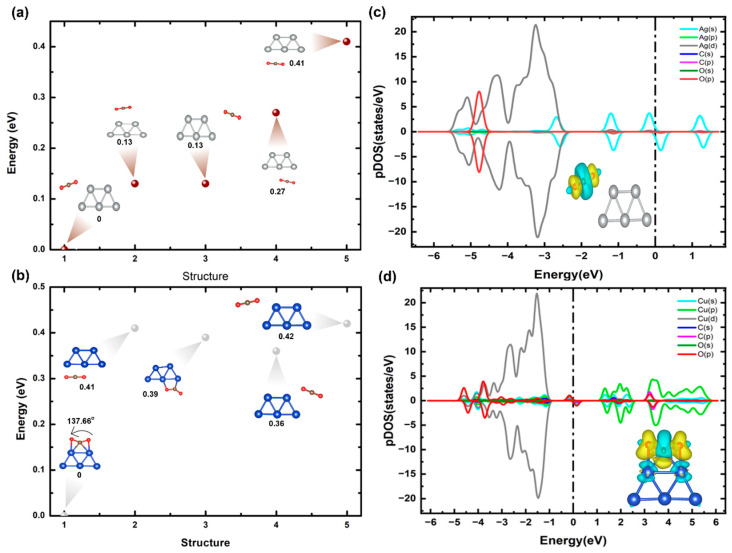
(**a**) Relative energies of optimized Ag_5_@CO_2_ bimetallic clusters by varying sites of Ag atom. (**b**) Relative energies of optimized Cu_5_@CO_2_ bimetallic clusters by varying sites of Cu atom. (**c**) pDOS and CDD of the Ag_5_@CO_2_ bimetallic cluster. (**d**) pDOS and CDD of the Cu_5_@CO_2_ bimetallic cluster. Blue and gray balls denote copper and silver atoms, respectively.

Bimetallic clusters have gathered substantial interest due to their remarkable catalytic activity and reduced susceptibility to CO_2_ poisoning [44]. The primary aim of this research is to understand the reactivity of CO_2_ gas molecules on CumAgn bimetallic clusters. Therefore, we explore the influence of compositional changes on the reactivity of CO_2_ molecules across bimetallic clusters of Cu_m_Ag_n_. Before adsorbing the CO_2_ molecule onto the Cu_m_Ag_n_ bimetallic clusters, we optimized the isolated CO_2_ molecule using GGA/DFT (see Figure A1 in Appendix A). The calculated bond length (d_C=O_ = 1.172 Å for CO_2_) and bond angle (θ_OCO_ = 179.96° for CO_2_) are in agreement with the findings of previous studies [45,46]. The initial phase of the catalytic conversion of CO_2_ involves the adsorption mechanism, where the molecule can either undergo physisorption or chemisorption onto the catalyst [26,45,47]. In the chemisorbed state, CO_2_ displays elongated C-O bonds and a decreased O-C-O bond angle, shifting from a linear to a bent configuration. This transformation implies that CO_2_ activation occurs due to electron transfer from the metal catalyst to the CO_2_ molecule’s π molecular orbitals [7,48]. Conversely, in the physisorbed state, CO_2_ retains its gas-phase characteristics, with an O-C-O bond angle of 180° and a C-O bond length of 1.18 Å. To provide a more systematic investigation, we carried out additional calculations on the Ag_5_ and Cu_5_ mono-clusters interacting with CO_2_, as shown in Figure A2. The relative energies of optimized Cu_5_@CO_2_ structures (Figure A2b) illustrate that structure 1 is the most stable, with an adsorption energy (0.70 eV) comparable to that of the Cu_4_Ag_1_@CO_2_ cluster. In contrast, the Ag_5_@CO_2_ configurations (Figure A2a) display a slightly different stability trend, with Structure 1 also being the most favorable but exhibiting different adsorption characteristics compared to Cu_5_@CO_2_. The pDOS and CDD plots for Ag_5_@CO_2_ and Cu_5_@CO_2_ are presented in Figure A2c,d, respectively. The pDOS for Ag_5_@CO_2_ signifies a less pronounced interaction between the Ag d-orbitals and CO_2_ at lower energy states (~−4.5 eV), while the Cu_5_@CO_2_ cluster reveals remarkable hybridization between the Cu d-orbitals and the CO_2_ orbitals in the range between −1 and −5 eV. This difference in electronic structure agrees with the CDD plots, where Cu_5_@CO_2_ exhibits more charge density transfer to the CO_2_ molecule than Ag_5_@CO_2_, similar to the trend observed in Cu_4_Ag_1_@CO_2_. Comparing these results with the Cu_4_Ag_1_@CO_2_ cluster, we observe that Cu_5_@CO_2_ possesses a higher charge transfer capacity, which could suggest enhanced CO_2_ activation. However, the mixed composition in Cu_4_Ag_1_@CO_2_ favors both stability and effective electron transfer, giving an ideal balance for CO_2_ activation. This systematic comparison emphasizes the role of Cu and Ag content in impacting the adsorption behavior and electronic attribution, aiding to enhance the design principles for efficient CO_2_ reduction catalysts.

**Figure 6:**
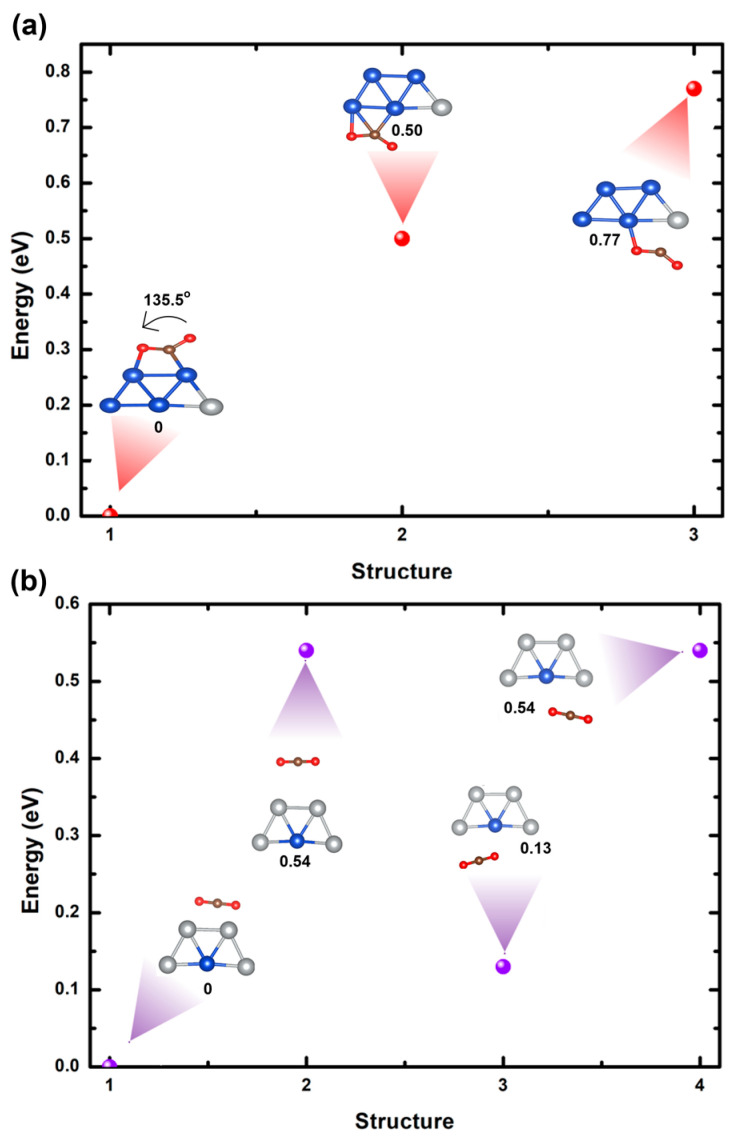
(**a**) Relative energies of optimized Cu_4_Ag_1_@CO_2_ bimetallic clusters by varying sites of CO_2_ molecules. (**b**) Relative energies of optimized Cu_1_Ag_4_@CO_2_ bimetallic clusters by varying sites of CO_2_ molecules. Blue, gray, red, and brown balls denote copper, silver, oxygen, and carbon atoms, respectively.

Considering the most stable configuration of the Cu_4_Ag_1_ bimetallic cluster, i.e., structure 1 in Figure 2a, we placed the CO_2_ molecule at different sites of the cluster as displayed in Figure 6a, and we found that structure 1 is the most stable configuration compared to the metastable state, specifically, structure 2, with an energy difference of roughly 0.5 eV. In structure 1, the CO_2_ is highly adsorbed on the bimetallic cluster with an adsorption energy of 0.95 eV, implying a chemisorption process. Our result showed that the systems responsible for CO_2_ activation consistently illustrate effective adsorption and significant charge transfer of around 0.6 e¯ from the Cu_4_Ag_1_ to the CO_2_ molecule. This process leads to the transformation of the CO_2_ molecule from a linear to a bent structure (θ_OCO_ = 135.5° for CO_2_), along with an elongation of the C-O bonds (d_O-C-O_ = 1.29, 1.24 Å), which is in good agreement with a previously published study on bimetallic CuNi nanoparticles [45]. The CO_2_ molecule favorably binds to the top sites of the Cu4Ag1 bimetallic cluster, forming bonds with two Cu atoms (d_O-Cu_ = 1.95 Å and d_C-Cu_ = 1.96 Å). The adsorption of CO_2_ on Cu_4_Ag_1_ bimetallic clusters results in modifications in the geometric structures of the clusters, with an observed Cu−Cu average bond length of approximately 2.41 Å. The outcomes indicate that bonding the CO_2_ to two Cu atoms in Cu_4_Ag_1_ bimetallic clusters can enhance their surface activity.

Figure 6b shows the adsorption of CO_2_ on bimetallic Cu_1_Ag_4_ clusters. In this case, the CO_2_ molecule exhibits a preference for the “top” site, yet no bonds form between CO_2_ and silver atoms. Considering the most stable configuration of the Cu_1_Ag_4_ bimetallic cluster as depicted in structure 1 in Figure 2b, we positioned the CO_2_ molecule at various sites on the cluster, as demonstrated in Figure 6b. Our estimates revealed that structure 1 remains the most stable structure, in contrast to the metastable state, structure 2, with an energy difference of nearly 0.13 eV. In structure 1, although the CO_2_ molecule exhibits high adsorption on the bimetallic cluster with an adsorption energy of 0.87 eV, the bond angles (θ_OCO_ = 179.67° for CO_2_) and bond lengths of CO_2_ (d_O=C=O_ = 1.18, 1.19 Å) closely resemble those found in isolated CO_2_. This could be explained by slight charge transfer to the CO_2_ molecule, which is nearly 0.03 e¯. The calculated average bond length of Ag-Ag was observed to be approximately 2.63 Å, which is in good agreement with the study of Ag_5_@CO_2_ [49]. It can be concluded that although the adsorption energies of CO_2_ are comparable for both systems, the degree of electron transfer to CO_2_ varies substantially, which distinguishes the process of CO_2_ activation. Further comparison of both systems’ geometric structural parameters is illustrated in Table 1.

**Figure 7:**
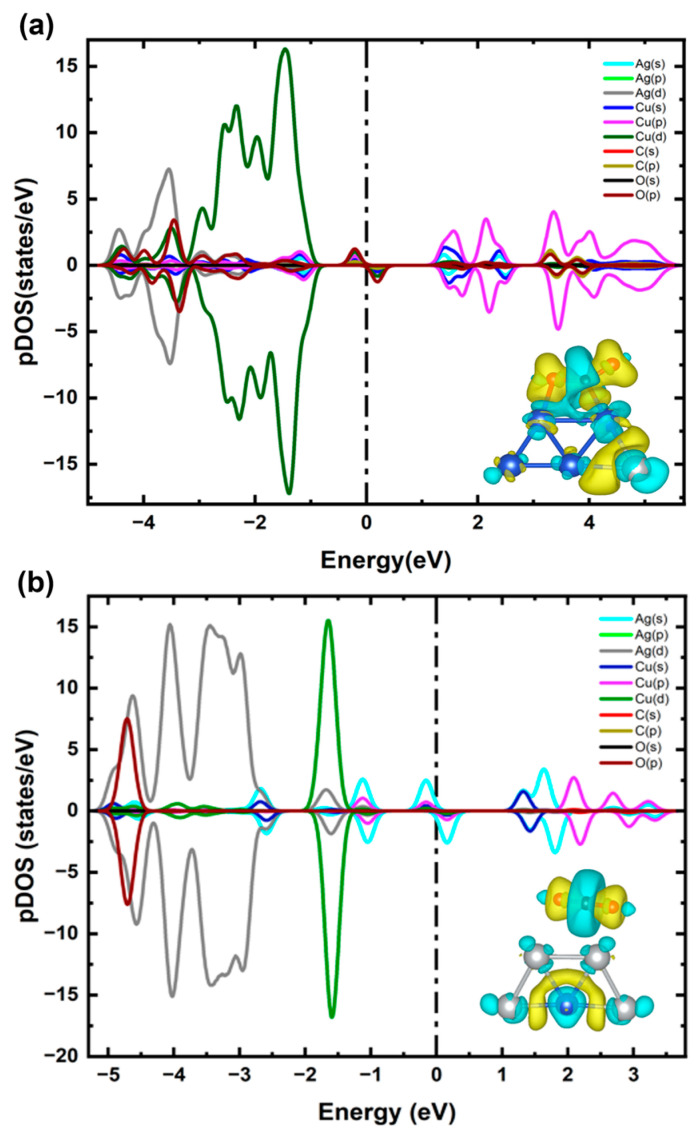
(**a**) pDOS and CDD of the Cu_4_Ag_1_@CO_2_ bimetallic cluster. (**b**) pDOS and CDD of the Cu_1_Ag_4_@CO_2_ bimetallic cluster.

To elucidate the factors affecting the chemisorption of CO_2_ on the Cu_4_Ag_1_ bimetallic cluster, we performed calculations to determine the pDOS as demonstrated in Figure 7a. As illustrated in Figure 7a and supported by reference [50], Cu_4_Ag_1_@CO_2_ exhibits delocalized orbitals around the Fermi level, which is clearly evident by the hybridization of sp orbitals of Cu and O. This overlap can facilitate substantial electron transfer from these clusters to CO_2_, which in turn stabilizes the chemisorbed state. In contrast, the overlap orbitals of Cu_1_Ag_4_ with O (p) orbitals of the CO_2_ molecule are characterized by considerably lower energy levels from the Fermi level, which can be observed at −4.7 eV in Figure 7b. Furthermore, our investigation into the electron excitation of Cu_4_Ag_1_ clusters shows that the energy level of the lowest unoccupied molecular orbital (LUMO) in CO_2_ closely matches the excitation energies of p electrons in Cu_4_Ag_1_ clusters (see states at 3.2 eV in Figure 7a). This alignment enables the transfer of excited hot electrons from the LUMO of Cu_4_Ag_1_ clusters to the LUMO of CO_2_ owing to the strong coupling between their orbitals. The resultant electron-transfer state possesses a chemisorption state, which drastically reduces the energy barriers for breaking the C-O bond and forming CO [51]. While in the Cu_1_Ag_4_ system, we observed no hybridized LUMO states of the CO_2_ molecule with the Cu_1_Ag_4_ cluster, implying a stable state of the CO_2_ molecule. In addition, the excess charge observed near the O atom signifies its predominant role in the adsorption of the CO_2_ molecule rather than the C atom [46]. Charge density maps, as shown in Figure 7a, corroborate our results regarding the electron transfer from the clusters to the CO_2_ molecule.

### 2.4. Adsorption of CO_2_ on Cu_3_Ag_2_ and Cu_2_Ag_3_ Clusters

**Figure 8:**
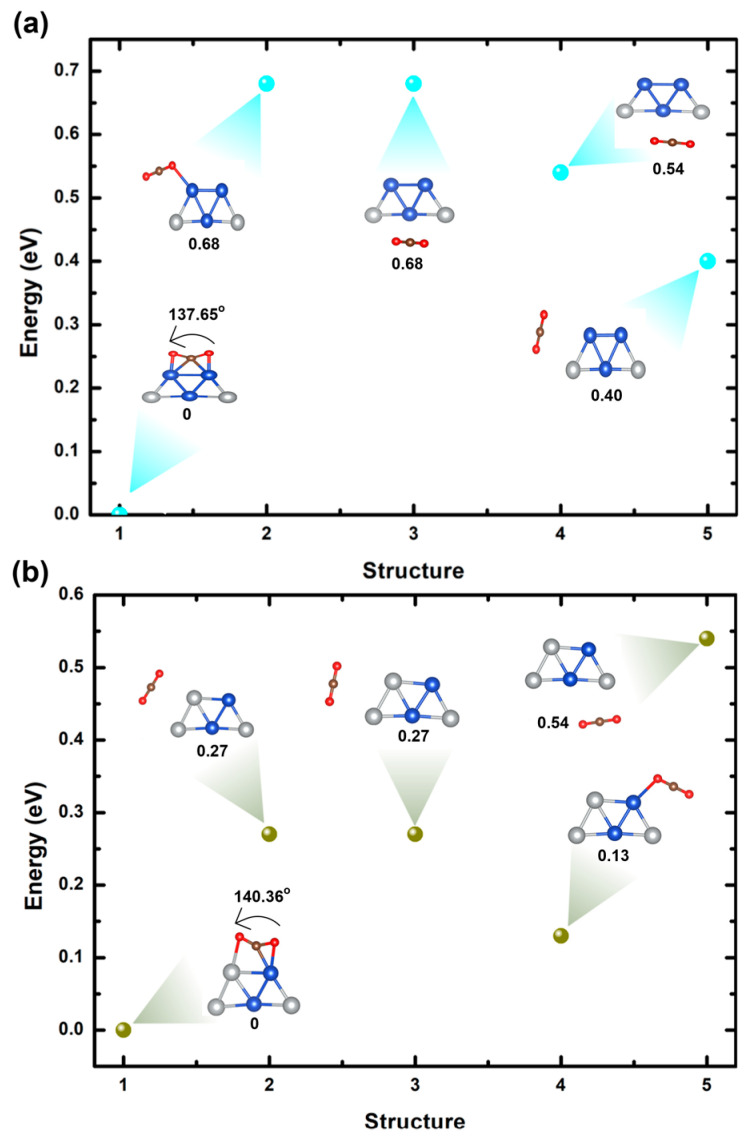
(**a**) Relative energies of optimized Cu_3_Ag_2_@CO_2_ bimetallic clusters by varying sites of CO_2_ molecules. (**b**) Relative energies of optimized Cu_2_Ag_3_@CO_2_ bimetallic clusters by varying sites of CO_2_ molecules.

Upon analyzing the most stable configuration of the Cu_3_Ag_2_ bimetallic cluster, structure 1 in Figure 4a, we explored the positioning of the CO_2_ molecule at various sites on the cluster, as shown in Figure 8a. Our calculation indicates that structure 1 represents the most stable cluster, with an energy difference of about 0.40 eV compared to the less stable structure 5. Structure 1 shows strong CO_2_ adsorption on the bimetallic cluster, with an adsorption energy of 0.81 eV, indicative of a chemisorption mechanism. Our results further demonstrate that systems involved in CO_2_ activation typically reveal strong adsorption and moderate electron transfer, approximately 0.14 e^−^, from the Cu_3_Ag_2_ bimetallic cluster to the CO_2_ molecule. This interaction induces a transformation in the CO_2_ molecule from a linear to a bent geometry (θ_OCO_ = 137.65° for CO_2_) and elongates the C-O bonds (d_O=C=O_ = 1.27, 1.26 Å). The CO_2_ molecule selectively binds to the sites on the Cu_4_Ag_1_ bimetallic cluster with the highest adsorption, forming chemical bonds with two Cu atoms (d_O-Cu_ = 2.10 Å and d_C-Cu_ = 2.07 Å). The adsorption of CO_2_ on the Cu_3_Ag_2_ cluster results in geometric alterations, with an average bond length of approximately 2.44 Å between Cu atoms. These results imply that the surface activity of Cu_3_Ag_2_ bimetallic clusters can be enhanced by the bonding of CO_2_ to two Cu atoms.

Figure 8b shows the adsorption process of CO_2_ on bimetallic Cu_2_Ag_3_ clusters. In this scenario, after examining the most stable arrangement of the Cu_2_Ag_3_ bimetallic cluster, which is referred to as structure 1 in Figure 4b, we considered the placement of the CO_2_ molecule at several locations on the cluster, as shown in Figure 8b. The result indicates that structure 1 is the most stable configuration, showing an energy difference of roughly 0.13 eV when compared to the less stable structure 4. Structure 1 exhibits a high adsorption for CO_2_, as evidenced by its strong adsorption with an energy of 0.81 eV, implying a chemisorption process. Our result also reveals that systems responsible for CO_2_ activation generally demonstrate substantial adsorption and a high level of electron transfer, around 0.53 e^−^, from the Cu_2_Ag_3_ bimetallic cluster to the CO_2_ molecule. This interaction causes the CO_2_ molecule to transition from a straight shape to a curved one (θ_OCO_ = 140.36° for CO_2_) and lengthens the C-O bonds (d_O=C=O_ = 1.24, 1.26 Å). The CO_2_ molecule exhibits a strong preference for binding to the top site on the Cu_2_Ag_3_ bimetallic cluster, making chemical bonds with one Cu atom and one Ag atom (with bond distances of d_O-Cu_ = 2.10 Å, d_O-Ag_ = 2.45 Å, and d_C-Cu_ = 2.03 Å). The results indicate that the surface reactivity of Cu_2_Ag_3_ bimetallic clusters can be improved through the bonding of CO_2_ to the top site of the cluster. Table 2 presents a detailed comparison of the geometric structural parameters of both systems.

**Figure 9:**
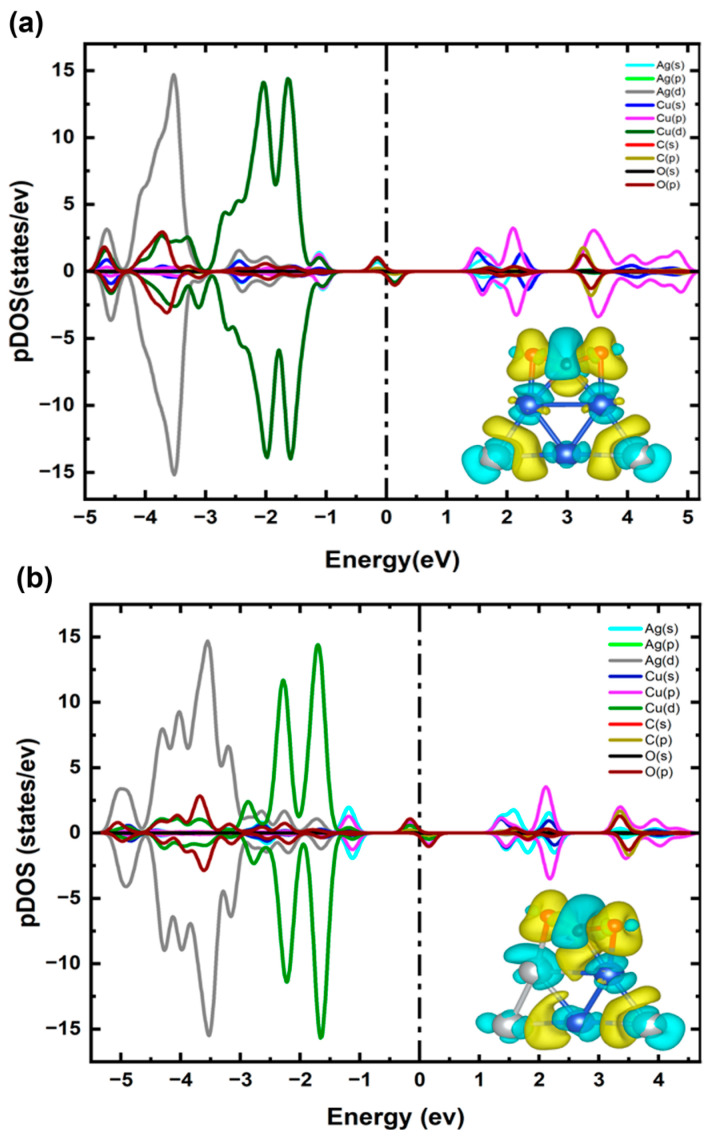
(**a**) pDOS and CDD of the Cu_3_Ag_2_@CO_2_ bimetallic cluster. (**b**) pDOS and CDD of the Cu_2_Ag_3_@CO_2_ bimetallic cluster.

To elucidate the factors influencing the chemisorption of CO_2_ on the Cu_3_Ag_2_ bimetallic cluster, we performed calculations to determine pDOS, as shown in Figure 9a. Figure 9a indicates that Cu_3_Ag_2_@CO_2_ exhibits delocalized states near the Fermi level, evidenced by the hybridization of Ag (s), Cu (d), and O (p), and C (p) orbitals. This overlapping orbital facilitates charge transfer from the cluster to CO_2_, thereby stabilizing the chemisorbed state. Similarly, the orbitals of the Cu_2_Ag_3_ cluster overlap with the CO_2_ orbitals, namely Cu (p), Cu (d), O (p), and C (p), as observed at Fermi energy in Figure 9b. This hybridization allows charge transfer from the cluster to CO_2_. Additionally, our analysis of electron excitation in Cu_3_Ag_2_ clusters shows that the energy level of the LUMO in CO_2_ closely matches the excitation energies of p electrons in Cu_3_Ag_2_ clusters (see states at 3.2 eV in Figure 9a). The alignment between the LUMO of Cu_4_Ag_1_ clusters and the LUMO of CO_2_ facilitates effective transmission of excited hot electrons due to strong orbital coupling. This charge-transfer state adopts a chemisorption structure, effectively lowering the energy barriers for C-O bond dissociation and CO formation. Similarly, in the Cu_1_Ag_4_ system, the energy level of the LUMO in CO_2_ aligns closely with the excitation energies of the p electrons of Cu and s electrons of Ag, particularly at the states around 3.5 eV, as illustrated in Figure 9b. This alignment between the LUMO of Cu_4_Ag_1_ clusters and the LUMO of CO_2_ enables the efficient transfer of excited hot electrons, supported by a strong orbital interaction. Furthermore, the presence of excess charge near the O atom suggests its predominant role in CO_2_ adsorption, as opposed to the C atom. The CDD maps presented in Figure 9a,b corroborate our results regarding electron transfer from the clusters to the CO_2_ molecule.

## 3. Computational Methodology

The calculations for trapezoidal Cu_m_Ag_n_ bimetallic clusters were conducted using the Kohn–Sham (KS) [52] spin-polarized DFT method, facilitated by the Quantum ESPRESSO software program v6.7 [53]. The exchange-correlation energies were assessed with the Perdew–Burke–Ernzerhof (PBE) functional [54], an implementation of the generalized gradient approximation (GGA) [55]. Electron-ion interactions were modeled with a projected augmented-wave (PAW) pseudopotential type [56]. A kinetic energy cut-off of 40 Ry was set to the plane-wave basis wave function, and a cut-off of 225 Ry was utilized for the charge density. To enhance convergence for bimetallic clusters, Gaussian smearing was utilized for the electronic levels. The optimization of all structures was carried out with a force convergence criterion set to 10^−3^ eV/Å, allowing all atoms to relax. During the process of geometric relaxation, a convergence threshold value of 10^−6^ Ry was used to achieve the electronic self-consistency. To prevent interactions between periodic images, the Cu_m_Ag_n_ bimetallic clusters were placed in a large supercell measuring approximately 17 Å × 14 Å × 10 Å for optimization. In the relaxation step, all calculations were performed at the Γ-point within the Brillouin zone. Then single-point calculations were performed, where a K-point grid of 2 × 2 × 2 was used to improve the accuracy of the electronic properties results. Furthermore, the Bader charge technique was utilized to investigate electron transfer [57]. The Visualization for Electronic and Structural Analysis (VESTA) software [58] was utilized to analyze CDD figures and to visualize all models presented in this study. We calculated the adsorption energy ($E_{ads}$) of CO_2_ using the following equation.

(1) $$E_{ads}=E_{tot}-E_{cluster}-E_{CO2}$$

where $E_{tot}$ is the system’s total energy, $E_{cluster}$ is the bimetallic cluster’s total energy, and $E_{CO2}$ is the isolated CO_2_ molecule’s total energy. The charge density difference was calculated using the following equation.

(2) $$\Delta_{\rho }=\rho_{ABC}-\rho_{A}-\rho_{B}$$

where $\rho_{ABC}$ is the charge density of the whole system and $\rho_{A}$ and $\rho_{B}$ are the charge density of system A and system B in the system, respectively.

## 4. Conclusions

This study presents a comprehensive analysis of the structural and electronic properties of the Cu_n_Ag_m_ bimetallic clusters, which offers valuable insights into the design of stable and efficient catalytic materials for CO_2_ conversion into useful products. Through a systematic investigation of Cu_m_Ag_n_ clusters (Cu_4_Ag_1_, Cu_1_Ag_4_, Cu_3_Ag_2_, and Cu_2_Ag_3_), we observed significant variations in electronic properties, stability, and CO_2_ interaction, influenced strongly by the specific arrangement of Cu and Ag atoms. The Cu_4_Ag_1_ cluster, featuring Ag at the bottom corners, demonstrated the highest stability due to enhanced bonding interactions, whereas the Cu_1_Ag_4_ configuration showed notable stability with Cu centered, facilitating efficient electron transfer from Cu to Ag, thereby enhancing catalytic potential.

In terms of CO_2_ adsorption, the Cu_4_Ag_1_ cluster exhibited the highest chemisorption energy and significant electron transfer to the CO_2_ molecule, leading to effective activation of CO_2_ for catalytic reactions. Although the Cu_1_Ag_4_ cluster displayed strong adsorption energy, it showed limited electron transfer, suggesting a less efficient activation process. Both Cu_3_Ag_2_ and Cu_2_Ag_3_ clusters also demonstrated strong adsorption energies and electron transfer capabilities, though with distinct structural distortions and bonding behaviors that underscore the impact of atomic composition and configuration on catalytic efficiency. Overall, this comparative analysis emphasizes the crucial role of atomic configuration in optimizing the catalytic properties of Cu_m_Ag_n_ bimetallic clusters for CO_2_ reduction. These findings underscore the potential for designing bimetallic clusters with targeted compositions and structures to enhance their catalytic performance in industrial CO_2_ reduction applications, advancing sustainable approaches for addressing critical environmental challenges.

## Figures and Tables

**Table 1:** Adsorption energy (E_ads_) of CO_2_, bond length (d_O=C=O_) of C-O, and bond angle (θ_OCO_) of the CO_2_ molecule. For reference, the calculated bond lengths and angles of the isolated CO_2_ molecule are 1.172 Å and 179.96 ^o^, respectively.

System	E_ads_ (eV)	d_O=C=O_ (Å)	θ_OCO_^(o)^
**Cu_4_Ag_1_**			
Structure 1	0.95	1.29, 1.24	135.50
Structure 2	0.54	1.28, 1.24	136.59
Structure 3	0.27	1.27, 1.22	139.65
**Cu_1_Ag_4_**			
Structure 1	0.87	1.18, 1.19	179.67
Structure 2	0.40	1.19, 1.19	179.65
Structure 3	0.81	1.19, 1.19	179.71
Structure 4	0.40	1.19, 1.19	179.50

**Table 2:** Adsorption energy (E_ads_) of CO_2_, bond length (d_O=C=O_) of C-O, and bond angle (θ_OCO_) of CO_2_ molecule.

System	E_ads_ (eV)	d_O=C=O_ (Å)	θ_OCO_^(o)^
**Cu_3_-Ag_2_**			
Structure 1	0.81	1.27, 1.26	137.65
Structure 2	0.13	1.18, 1.19	179.74
Structure 3	0.13	1.19, 1.19	179.68
Structure 4	0.27	1.19, 1.19	179.39
Structure 5	0.40	1.19, 1.18	179.89
**Cu_2_-Ag_3_**			
Structure 1	0.81	1.24, 1.26	140.36
Structure 2	0.54	1.19. 1.19	179.65
Structure 3	0.54	1.19, 1.18	179.79
Structure 4	0.68	1.19, 1.18	179.78
Structure 5	0.27	1.18, 1.19	179.06

## Data Availability

Data are contained within the article.

## Appendix A

This section contains calculations on the isolated CO_2_ molecule, such as the optimized structure, net charge, charge density difference, and partial density of states.
